# Diversity of Pectin Rhamnogalacturonan I Rhamnosyltransferases in Glycosyltransferase Family 106

**DOI:** 10.3389/fpls.2020.00997

**Published:** 2020-07-02

**Authors:** Bussarin Wachananawat, Takeshi Kuroha, Yuto Takenaka, Hiroyuki Kajiura, Satoshi Naramoto, Ryusuke Yokoyama, Kimitsune Ishizaki, Kazuhiko Nishitani, Takeshi Ishimizu

**Affiliations:** ^1^ College of Life Sciences, Ritsumeikan University, Kusatsu, Japan; ^2^ Graduate School of Life Sciences, Tohoku University, Sendai, Japan; ^3^ Ritsumeikan Global Innovation Research Organization, Ritsumeikan University, Kusatsu, Japan; ^4^ Faculty of Science, Hokkaido University, Sapporo, Japan; ^5^ Graduate School of Science, Kobe University, Kobe, Japan; ^6^ Faculty of Science, Kanagawa University, Hiratsuka, Japan

**Keywords:** glycosyltransferase, GT106, *Marchantia polymorpha*, pectin, rhamnogalacturonan I, rhamnosyltransferase

## Abstract

Rhamnogalacturonan I (RG-I) comprises approximately one quarter of the pectin molecules in land plants, and the backbone of RG-I consists of a repeating sequence of [2)-α-L-Rha(1-4)-α-D-GalUA(1-] disaccharide. Four *Arabidopsis thaliana* genes encoding RG-I rhamnosyltransferases (AtRRT1 to AtRRT4), which synthesize the disaccharide repeats, have been identified in the glycosyltransferase family (GT106). However, the functional role of RG-I in plant cell walls and the evolutional history of RRTs remains to be clarified. Here, we characterized the sole ortholog of AtRRT1–AtRRT4 in liverwort, *Marchantia polymorpha*, namely, MpRRT1. MpRRT1 had RRT activity and genetically complemented the At*RRT1*-deficient mutant phenotype in *A. thaliana*. However, the Mp*RRT1*-deficient *M. polymorpha* mutants showed no prominent morphological changes and only an approximate 20% reduction in rhamnose content in the cell wall fraction compared to that in wild-type plants, suggesting the existence of other *RRT* gene(s) in the *M. polymorpha* genome. As expected, we detected RRT activities in other GT106 family proteins such as those encoded by Mp*RRT3* in *M. polymorpha* and *FRB1/*At*RRT8* in *A. thaliana*, the deficient mutant of which affects cell adhesion. Our results show that *RRT* genes are more redundant and diverse in GT106 than previously thought.

## Introduction

Rhamnogalacturonan I (RG-I), along with homogalacturonan (HG) and RG-II, constitutes the major part of the pectin in cell walls of land plants, which include charophytes, bryophytes, and vascular plants (Kulkarni et al., 2012; O’Raurke et al., 2015). RG-I consists of a ramified backbone composed of the disaccharide repeating unit [2)-α-L-Rha(1-4)-α-D-GalUA(1-] (Lau et al., 1985), which is branched at the *O*4 or *O*3 position of Rha residues with arabinan, galactan, or arabinogalactan (Lau et al., 1987). Some of these side chains are further modified by fucose and glucose residues at the site close to the backbone (Nakamura et al., 2001) or terminal ferulic acid (Ishii, 1997; Ralet et al., 2005).

The contents and/or branching pattern of RG-I depend on the plant species, tissues of the same plants, and developmental stages of the same tissues. The side chains of RG-I are developmentally or spatially regulated in pea cotyledons (McCartney et al., 2000), *Arabidopsis thaliana* inflorescence stems (Phyo et al., 2017), G-layers in poplar tension wood (Gorshkova et al., 2015; Guedes et al., 2017), seed mucilage (Dean et al., 2007; Macquet et al., 2007), and fruit softening (Orfila et al., 2002; Paniagua et al., 2016; Wang et al., 2019). These studies showed that RG-I is related to the maturation and mechanical properties of some tissues; however, the associated molecular mechanisms remain unclear. The functional roles of pectin in cell wall mechanics are considered more important than previously thought (Cosgrove, 2018; Haas et al., 2020); however, functional analyses of RG-I are insufficient to determine its roles because appropriate RG-I-deficient mutants are not easily created.

To address the functional roles of RG-I polysaccharides, it is vital to identify and analyze corresponding biosynthetic genes. An analysis of *A. thaliana* mutants deficient in *ARABINAN DEFICIENT 1* (*ARAD1*) and/or *ARAD2*, which are responsible for arabinan elongation, previously revealed that arabinan has a role in the mechanical properties of inflorescence stems (Harholt et al., 2006; Verherbruggen et al., 2013). Further, loss-of-function mutants in *GALACTAN SYNTHASE 1* (*GALS1*) to *GALS3*, which are involved in galactan elongation, do not show any obvious phenotypic change, and analyses of their double or triple deficient mutants have not been reported to date (Liwanag et al., 2012; Ebert et al., 2018). Four backbone-synthetic RG-I rhamnosyltransferases (AtRRT1 to AtRRT4) in the glycosyltransferase family (GT 106) were recently identified in *A. thaliana* (Takenaka et al., 2018). The At*RRT1*-deficient mutant was observed to have reduced content of RG-I in seed mucilage; however, the RG-I-deficient mutant (deficient in all four At*RRT* genes) has not been analyzed to date. Thus, it is not easy to prepare RG-I-deficient mutants and analyze the functions of RG-I polysaccharides because the genes encoding its biosynthetic enzymes are redundant.

The genes encoding cell wall polysaccharide biosynthetic-enzymes of vascular plants are also found in bryophytes and charophytes (Mikkelsen et al., 2014; Bowman et al., 2017), suggesting that typical cell walls of land plants are required for plant terrestrialization. Among the various plant species, *Marchantia polymorpha*, a liverwort in bryophyte, is an attractive plant species for the functional analysis of cell walls because it exhibits low genetic redundancy compared to other land plants (Bowman et al., 2017). Bryophyte plants also have RG-I in their cell walls (Kulkarni et al., 2012; Roberts et al., 2012; McCarthy et al., 2014). Vascular plants including *A. thaliana* have two or more RG-I-synthetic *RRT* genes in their genomes, whereas the *M. polymorpha* genome has only one gene homologous to At*RRT* genes (Bowman et al., 2017; Takenaka et al., 2018). An analysis of the *M. polymorpha* mutant deficient in this gene is an attractive approach to elucidate the functional and evolutionary roles of RG-I in the cell wall, even though its life cycles, reproduction system, and the presence of xylem are to some extent distinct from those of vascular plants. The biochemical and functional analyses of *RRT* in *M. polymorpha* in this study were performed to provide profound knowledge of *RRT* genes in land plants, including their evolutionary history and gene redundancy in plant genomes.

## Materials and Methods

### Plant Materials

The *A. thaliana* T-DNA insertion lines for At*rrt1* (SALK_022924 and SALK_042968) were obtained from the Arabidopsis Biological Resource Center. The seeds of wild-type (Col-0) and At*rrt1* strains were grown on MS medium at 22°C under a 16-h light/8-h dark cycle. After 10 days, the seedlings were transferred to compounded soil under the same conditions. Homozygous insertion mutant lines were identified by PCR analysis (Takenaka et al., 2018). The *Nicotiana benthamiana* seeds were sown in the soil and incubated with a 16-h light/8-h dark cycle at 22°C for 2 weeks. The seedlings were transferred to each pot under the same conditions for 6 weeks.

A male accession of liverwort (*M. polymorpha*), Takaragaike-1 (Tak-1), was used in this study and asexually maintained *via* the transplantation and growth of gemmae (Ishizaki et al., 2008). Liverworts were cultured on a Petri dish using half-strength Gamborg’s B5 (1/2 B5) medium (Gamborg et al., 1968) containing 1% agar (Nacalai Tesque), under continuous 50 to 60 μmol/m^2^·s white fluorescent light at 22°C. To induce the reproductive phase, thalli were transferred to plastic cases with 1/2 B5 medium containing 1% agar and grown under continuous 50 to 60 μmol/m^2^·s white light supplemented with 10 to 20 μmol m^−2^ s^−1^ far-red light irradiation at 22°C.

### Complementation of the At*rrt1-1* Mutant With Mp*RRT1*

The cDNA of the Mp*RRT1* (Mapoly0033s0138.1) gene was amplified by PCR with specific primers, MpRRT1_F and MpRRT1_R (**Supplementary Table 1**), using *M. polymorpha* cDNA as a template, and the resulting PCR product was cloned into the pWAT202 vector (Kumakura et al., 2013) using the in-fusion HD cloning kit (Clontech). The resulting construct was transformed into the At*rrt1-1* (SALK_022924) mutant *via Agrobacterium tumefaciens* (GV3101 strain)–mediated transformation (Holsters et al., 1978). Transgenic plants were selected on MS agar plates containing 10 μg/ml bialaphos. The seed mucilage phenotype was observed with a stereo microscope (Olympus MVX10). The mature dry seeds were soaked in water for 2 h and stained with 0.01% ruthenium red solution for 1 h and the color was washed out with water (McFarlane et al., 2014). The volume of seed mucilage was calculated by assuming that the seeds or seeds containing mucilage were spheroid. The length of each axis was calculated using ImageJ software.

### Expression of Recombinant RRTs

The full-length Mp*RRT1* and Mp*RRT3* (Mapoly0014s0149.1) open reading frames were amplified with sets of gene-specific primers, [MpRRT1-FLAG_F and MpRRT1-FLAG_R] and [MpRRT3-FLAG_F and MpRRT3-FLAG_R] (**Supplementary Table 1**), respectively, using *M. polymorpha* cDNA as a template. The full length *FRB1*/At*RRT8* open reading frame was amplified with gene-specific primers, AtRRT8-FLAG_F and AtRRT8-FLAG_R (**Supplementary Table 1**), using *A. thaliana* cDNA as a template. The amplified DNA was cloned into the pBI121 vector using the in-fusion HD cloning kit (Clontech). The recombinant *RRT* plasmids or the empty pBI121 vector were transformed into *A. tumefaciens* GV3101 strain using a freeze-thaw method (Holsters et al., 1978). The transformed *A. tumefaciens* were selected on an LB agar plate containing 50 µg/ml kanamycin, 50 µg/ml gentamycin, and 50 µg/ml rifampicin. The transformed *A. tumefaciens* cells (OD_600_ 0.5) were suspended in 10 mM MES-KOH buffer, pH 5.8, containing 10 mM MgSO_4_ and inoculated into *N. benthamiana* leaves *via* the vacuum infiltration method (Leuzinger et al., 2013). After infiltration, the plants were grown under 16-h light/8-h dark conditions at 22°C for 3 days. The tobacco leaves were ground with liquid nitrogen and homogenized with 25 mM Tris-HCl buffer, pH 7.0, containing 10 mM MgCl_2_, 2 mM dithiothreitol, 250 mM sucrose, 2 µM leupeptin, and 0.1 mM PMSF. The homogenate filtered through miracloth was centrifuged at 3,000 × *g* for 10 min at 4°C. The supernatant was centrifuged at 100,000 × *g* for 1 h at 4°C to pellet the microsomal fraction. The microsomal proteins (approximately, 1.2 mg) were solubilized in 100 µl of the buffer containing 50 mM Tris-HCl, pH 7.5, 150 mM NaCl, and 1.0% Triton X-100. The solubilized proteins were suspended with 10 µl of an anti-FLAG M2 affinity gel (Sigma-Aldrich) on a rotating wheel at 4°C for 1 h. The gel beads were then washed three times with 50 mM Tris-HCl buffer, pH 7.5, containing 150 mM NaCl and 0.01% Triton X-100. The amount of protein used for enzyme assays was approximately 1.5 µg. The recombinant proteins were detected by western blotting with a monoclonal anti-FLAG M2 antibody conjugated with alkaline phosphatase (1:2,000; Sigma-Aldrich) using Immobilon Forte Western HRP Substrate (Merck Millipore). Affinity-bound proteins from the microsomal fraction of tobacco leaves infiltrated with the empty pBI121 vector were used as a control.

### Assay for RG-I Rhamnosyltransferase

The oligosaccharides derived from RG-I were labeled with 2-aminopyridine at their reducing ends (Uehara et al., 2017) and used as acceptor substrates of RG-I rhamnosyltransferase. The structures and abbreviations for these oligosaccharides are listed in **Supplementary Table 2**. UDP-rhamnose was enzymatically synthesized as described previously (Ohashi et al., 2016). The RG-I rhamnosyltransferase assay was carried out in a reaction mixture (total volume, 10 µL) containing the enzyme, 50 mM HEPES-KOH buffer, pH 7.0, 25 mM KCl, 0.2 M sucrose, 0.05% bovine serum albumin, 0.25% Triton X-100, 0.5 mM UDP-Rha, and 50 μM GR_8_-PA at 30°C for 1 h. The reaction was terminated by heating at 100°C for 3 min. The enzyme product was separated using a TSKgel-DEAE-5PW column (7.5 mm × 75 mm) as described previously (Uehara et al., 2017). It was then detected based on its fluorescence (Ex 320 nm, Em 400 nm) and quantified from the peak area on the chromatogram.

### Functional Analyses of Mp*RRT1* in Liverwort

The sequence information of the *M. polymorpha* genome portal site MarpolBase (http://marchantia.info; JGI 3.1) was used for plasmid construction. Constructs for the CRISPR/Cas9-target mutagenesis of Mp*RRT1* were generated by annealing oligonucleotide pairs with MpRRT1_CR1_F and MpRRT1_CR1_R (**Supplementary Table 1**) and the ligation of these annealed products into the *Bsa*I site of a pMpGE_En03 vector (Addgene) (Sugano et al., 2018) to produce plasmids En03_Mp*RRT1*_CR1 including gRNA expression cassettes. The sequence between attL1 and attL2 within En03_Mp*RRT1*_CR1 was inserted into the binary vector pMpGE011 (Addgene) (Sugano et al., 2018) *via* an LR reaction using Gateway LR Clonase II Enzyme Mix (Thermo Fisher Scientific). The resulting construct, GE011_Mp*RRT1*_CR1, was introduced into Tak-1 using *A. tumefaciens* strain GV2260 as described previously (Kubota et al., 2013).

To construct reporter plasmids, a DNA fragment of the 5′-putative Mp*RRT1* promoter sequence was amplified by PCR using Prime STAR MAX DNA polymerase (Takara Bio) with MpRRT1-4217_F and MpRRT1_0_R primers (**Supplementary Table 1**) from the Tak-1 genome. The fragment was cloned into a pENTR/D-TOPO entry vector (Thermo Fisher Scientific). We found that the tandem duplication of a 1.0-kbp sequence in the Mp*RRT1* promoter did not exist in the PCR product sequence based on the results of Sanger sequencing and the fragment size of the cloned vector digested with the restriction enzyme *Nco*I. Therefore, we used this 3,217-bp fragment of the Mp*RRT1* promoter for further analyses. The resulting construct, pENTR_proMpRRT1, was inserted into the destination vectors pMpGWB316 and pMpGWB304 (Addgene) (Ishizaki et al., 2015) *via* the LR reaction using LR Clonase II Enzyme Mix to produce *pro*Mp*RRT1*:*tdTOMATO* and *pro*Mp*RRT1:GUS* plasmids, respectively.

For subcellular localization analysis of the MpRRT1-tagRFP fusion protein, the cDNA sequence of Mp*RRT1* without a stop codon (TGA) was amplified by PCR using Prime STAR MAX DNA polymerase (Takara Bio) with MpRRT1_1_F and MpRRT1+3107_nostop_R primers (**Supplementary Table 1**), followed by cloning into the pENTR/D-TOPO entry vector. The Mp*RRT1* cDNA sequence of the resulting construct, pENTR_cMpRRT1ns, was inserted into the destination vector pMpGWB328 (Addgene) (Ishizaki et al., 2015) *via* an LR reaction to produce the *pro35S*:Mp*RRT1-tagRFP* plasmid. The plasmids *pro35S:*Mp*USE1A-Citrine*, *pro35S:MpSYP3-Citrine*, and *pro35S:*Mp*SYP4-Citrine* were obtained from Ueda, T. and Kanazawa, T. (National Institute for Basic Biology) (Kanazawa et al., 2016).

### Transformation of Liverwort

The transformation of liverwort using regenerating thalli was performed according to previously described methods (Kubota et al., 2013). Transformants were selected on plates containing 0.5 µM chlorsulfuron or 10 µg/ml hygromycin. For analyses of the subcellular localization of the MpRRT1-tagRFP protein, *pro35S:*Mp*USE1A-Citrine*, *pro35S:*Mp*SYP3-Citrine*, and *pro35S:*Mp*SYP4-Citrine* constructs were first transformed into Tak-1 thalli, followed by selection with hygromycin. Transformants were confirmed by observing Citrine fluorescence with a laser scanning confocal microscope (FV1000-D BX61, Olympus). Each established line was used for the second transformation with *pro35S:*Mp*RRT1-tagRFP* and selection with chlorsulfuron.

### Genomic DNA Extraction and Genotyping of Liverwort

For plasmid construction, the Tak-1 DNA was extracted from thalli using the DNeasy Plant Mini Kit (Qiagen) according to the manufacturer’s protocol. For genotyping after genome editing, DNA was extracted based on the following method. Approximately 5-mm^2^ thallus samples were grinded with a Tissuelyser II (Qiagen) with 150 µl of DNA extraction buffer [1.5 M Tris-HCl (pH 8.8), 10% (w/v) SDS, 10 M LiCl, 1.5 M EDTA (pH8.0)], and 150 µl phenol:chloroform:isoamyl alcohol (25:24:1) for 5 min at 20 Hz. Resulting lysates were centrifuged at 20,000 × *g* for 10 min at 4°C. The supernatant was mixed with the same volume of isopropanol. After another centrifuge step (20,000 × *g*; 10 min; 4°C), the supernatant was removed and the pellet was washed with 160 µl of 70% ethanol. After further centrifuging (20,000 × *g*; 10 min; 4°C), the supernatant was removed and the pellet was dried at room temperature for at least 5 min. The dried pellet was dissolved in 50 µl TE buffer and used as the DNA sample for genotyping. For PCR reactions, Tks Gflex DNA Polymerase (Takara Bio) was used according to the manufacturer’s protocol. Primers for genotyping after genome editing are described in **Supplementary Table 1**.

### Microscopy

The morphology of 9-day-old thalli and GUS-stained thalli was imaged using a stereomicroscope (Leica M205 FA). For reporter analyses with *pro*Mp*RRT:tdTOMATO*, tdTOMATO was excited at 546 nm under a fluorescence microscope (Leica DM5500 B). Confocal laser scanning microscopy observations were conducted using a Zeiss LSM880. Citrine and tagRFP were excited with 488-nm and 543-nm laser wavelengths, respectively. Samples were observed with LD-LCI Plan-Apochromat 40x/1.2 NA multi-immersion objectives.

### GUS Staining

Histochemical assays for GUS activity were performed as described previously (Ishizaki et al., 2012).

### Extraction and Analysis of Cell Wall Monosaccharides

For cell wall monosaccharide analysis, the cell wall samples were fractionated as described previously with minor modifications (Nishitani and Masuda, 1979). The 0- to 5-mm region from the apical notch of 3-week-old thalli were subjected to extraction with 80% ethanol at 80°C and a methanol/chloroform mixture at 25°C. Samples were next washed with 100% ethanol three times, followed by three washes with acetone. After removing acetone, the samples were hydrolyzed with 2 N trifluoroacetic acid for 1 h at 121°C. After evaporation and subsequent dissolution in water, monosaccharides were analyzed using a high-performance anion exchange chromatograph (DionexICS 5000) equipped with a CarboPak PA1 column. Detailed conditions for this analysis were described previously (Takenaka et al., 2018).

## Results

### *RRT* Gene in *M. polymorpha*

The *A. thaliana* genome possesses 34 genes predicted to encode glycosyltransferases of GT106 proteins. Four of the 34 genes (At*RRT1* to At*RRT4*) have been identified as encoding RRT enzymes responsible for RG-I synthesis (Takenaka et al., 2018). In contrast, *M. polymorpha* of the bryophytes possesses 13 genes predicted to encode GT106 proteins, and only a single *M. polymorpha* gene (Mapoly0033s0138.1) was found to be homologous to the four At*RRTs*. This gene was named Mp*RRT1* and further characterized in this study.

### MpRRT1 Has RG-I Rhamnosyltransferase Activity

**Figure 1:**
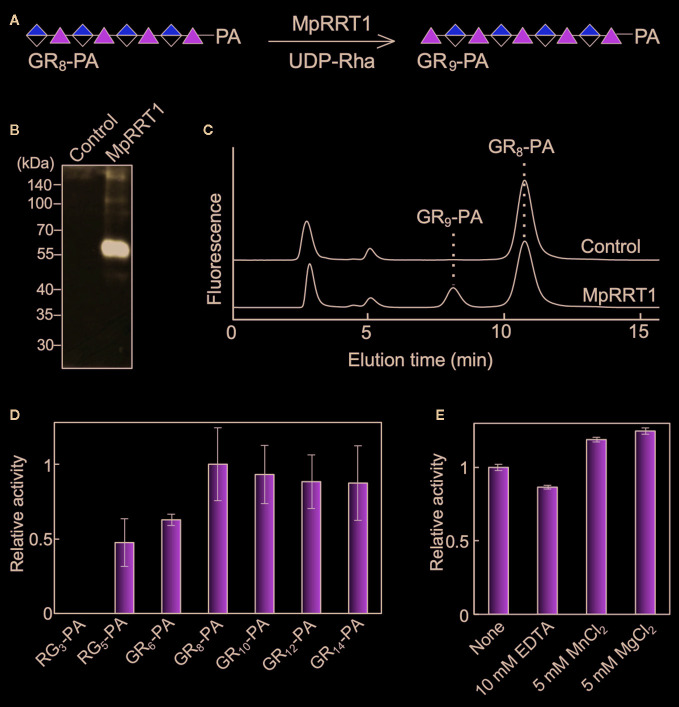
MpRRT1 has RG-I rhamnosyltransferase activity. **(A)** Reaction scheme for MpRRT1. Green triangles and yellow-divided diamonds represent Rha and GalUA residues, respectively. The fluorescent pyridylamino group (PA) is covalently linked to the reducing-end of the oligosaccharides. **(B)** SDS-PAGE of the recombinant MpRRT1 protein with a C-terminal FLAG tag expressed in tobacco leaves. The protein was detected by immunoblotting using an anti-FLAG antibody. The control proteins were prepared from tobacco leaves infiltrated with the empty pBI121 vector with the same procedure as that used to prepare MpRRT1. **(C)** Rhamnosyltransferase activity of MpRRT1. The recombinant protein was reacted with 50 µM GR_8_-PA and 0.5 mM UDP-Rha at 30°C for 1 h. The upper and lower chromatograms represent after the enzyme reaction with the fraction from control proteins and MpRRT1, respectively. The enzyme product GR_9_-PA was detected after the enzyme reaction with MpRRT1. **(D)** Acceptor substrate specificity of MpRRT1. The structures of acceptor oligosaccharides are shown in **Supplementary Table 2**. **(E)** Divalent cation-dependence of MpRRT1. Values are presented as the mean of three biologically independent samples with SD.

To examine RG-I rhamnosyltransferase activity of MpRRT1 using the fluorescent-labeled RG-I oligosaccharide (**Figure 1A**), we expressed recombinant MpRRT1 as a FLAG-tagged fusion protein in *N. benthamiana* leaves and purified it with an anti-FLAG M2 affinity gel. The recombinant protein was detected by immunoblotting as a 61-kDa protein, corresponding to its calculated molecular mass (61 kDa; **Figure 1B**). A 1-h reaction of the recombinant protein with the RG-I oligosaccharide, GR_8_-PA (**Supplementary Table 2**), and UDP-Rha quantitatively produced GR_9_-PA, a rhamnosyl residue-adduct of GR_8_-PA (**Figure 1C**), whereas the protein fraction prepared by the same procedure from *N. benthamiana* leaves transformed with the empty vector had no enzyme activity. These results show that MpRRT1 exhibits RG-I rhamnosyltransferase activity, acts on RG-I oligosaccharides with a degree of polymerization (DP) more than 5 as acceptor substrates, and prefers those with DP more than 8 (**Figure 1D**). The enzyme activity did not depend on divalent cations (**Figure 1E**). Its optimum pH and temperature were approximately 7.0 and 20°C, respectively, under the conditions used in this study. These characteristics are similar to those of AtRRTs (Takenaka et al., 2018).

### Mp*RRT1* Complements the At*rrt1-1* Mutant

**Figure 2:**
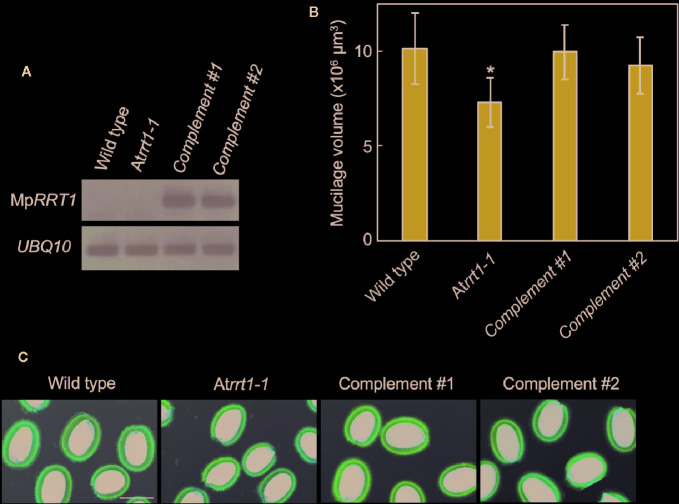
Mp*RRT1* complements the reduction in seed mucilage production in the At*rrt1-1* mutant. **(A)** RT-PCR analysis of the Mp*RRT1* transcript in the wild-type, At*rrt1-1* (SALK_022924), and two complemented At*rrt1-1* (*pro35S:*Mp*RRT1*/At*rrt1-1*) strains. *UBQ10* was used as a loading control. **(B)** Mucilage volumes of seed coats in the wild-type, At*rrt1-1*, and two complemented At*rrt1-1* strains. The mucilage volumes are presented as the mean values of 20 seeds with SD. The asterisk indicates that the value was significantly different from those of the wild-type and complemented At*rrt1-1* strains by Student’s t-test: *P < 0.05. **(C)** *Arabidopsis* seed mucilage of the wild-type, At*rrt1-1*, and two complemented At*rrt1-1* strains, stained with ruthenium red. Scale bar, 250 μm.

To functionally characterize Mp*RRT1 in planta*, we next investigated whether the overexpression of Mp*RRT1* cDNA complements the *A. thaliana* loss-of-function mutant, in which T-DNA was inserted into the At*RRT1* gene (**Figure 2**). Whereas the At*rrt1* mutants exhibited an approximate 28% reduction in seed mucilage volume (**Supplementary Figure S1** and **Figures 2B, C**; Takenaka et al., 2018), the *pro35S:*Mp*RRT1* transgenic lines in the At*rrt1-1* mutant background showed restored reductions in mucilage production characteristic of the At*rrt1-1* mutant (**Figures 2B, C**). These results suggest that MpRRT1 functions in the production of RG-I *in planta*, as is the case with AtRRT1 (Takenaka et al., 2018).

### Expression Profiles of Mp*RRT1* in Liverwort

**Figure 3:**
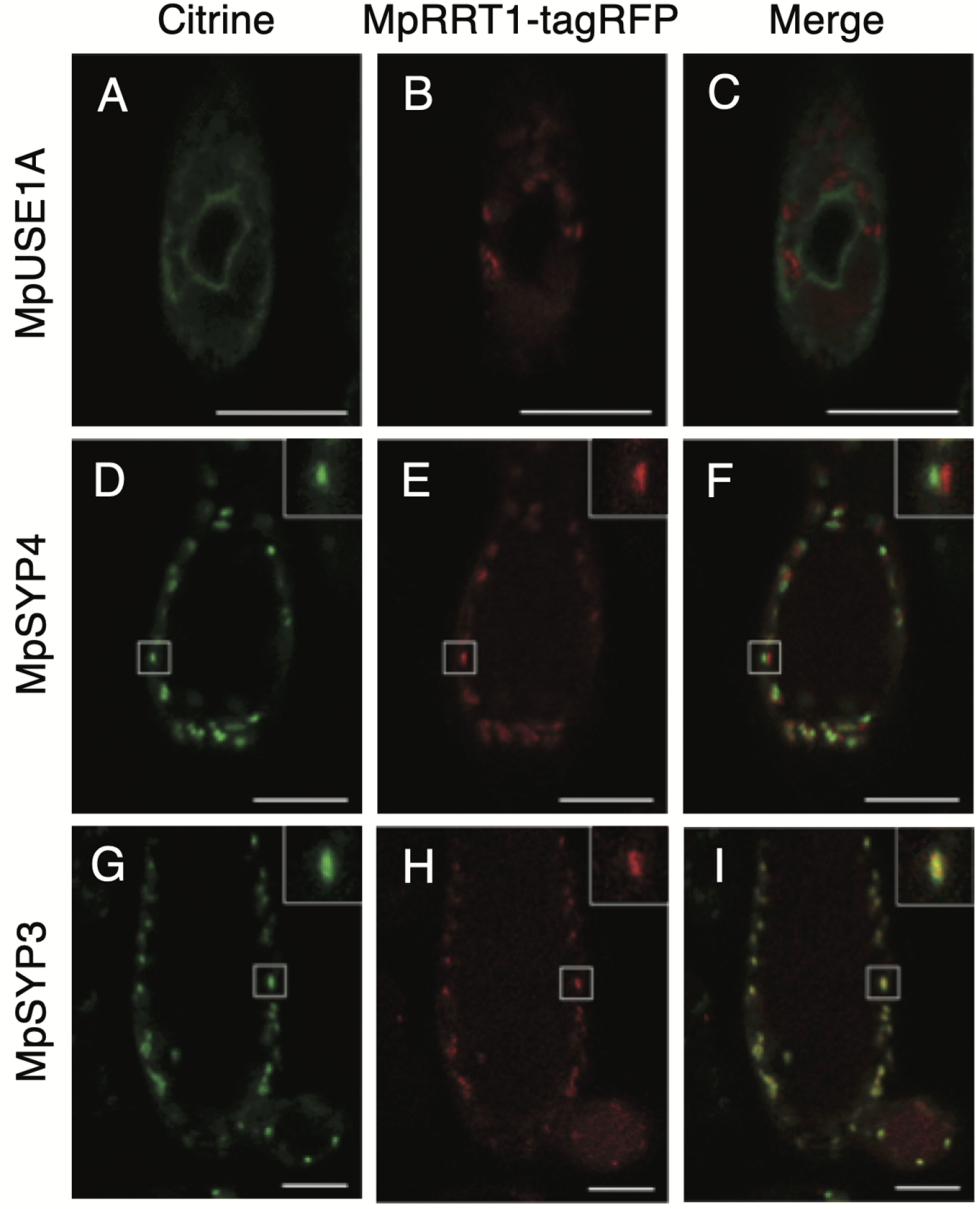
Subcellular localization of MpRRT1-tagRFP protein in liverwort. Thallus cells in 2-week-old gemmalings of transgenic *pro35S:*Mp*RRT1*-*tagRFP* liverworts co-overexpressing Citrine-MpUSE1A **(A–C)**, Citrine-MpSYP4 **(D–F)**, or Citrine-MpSYP3 **(G–I)** were observed with a laser scanning confocal microscope. The localization of the Citrine-proteins (green) **(A, D, G)**, tagRFP florescence **(B, E, H)**, and their merged images **(C, F, I)** are shown. In D–I, small white squares are shown at an increased magnification at the upper right. Scale bars, 25 μm.

Pectin biosynthesis occurs in the Golgi apparatus and AtRRT1 has been shown to localize to this organelle (Takenaka et al., 2018). We next compared the subcellular localization of MpRRT1 with those of marker proteins MpUSE1A, MpSYP4, and MpSYP3, of which the localizations are the endoplasmic reticulum, *trans*-Golgi network, and *cis*-Golgi, respectively (Kanazawa et al., 2016). Each marker protein fused with Citrine (green) was co-transformed with *pro35S*:Mp*RRT1-tagRFP* into thalli of liverworts. The red fluorescence of MpRRT1-tagRFP was distributed in a dot-like pattern (**Figures 3B, E, H**). This localization did not correspond to that of Citrine-MpUSE1A (**Figures 3A, C**). The dot-like patterns were also observed for Golgi-localized proteins, specifically Citrine-MpSYP4 (**Figure 3D**) and Citrine-MpSYP3 (**Figure 3G**). Among them, Citrine-MpSYP3 co-localized with the MpRRT1-tagRFP (**Figure 3I**), indicating that MpRRT1 localizes at the *cis*-Golgi in liverwort.

**Figure 4:**
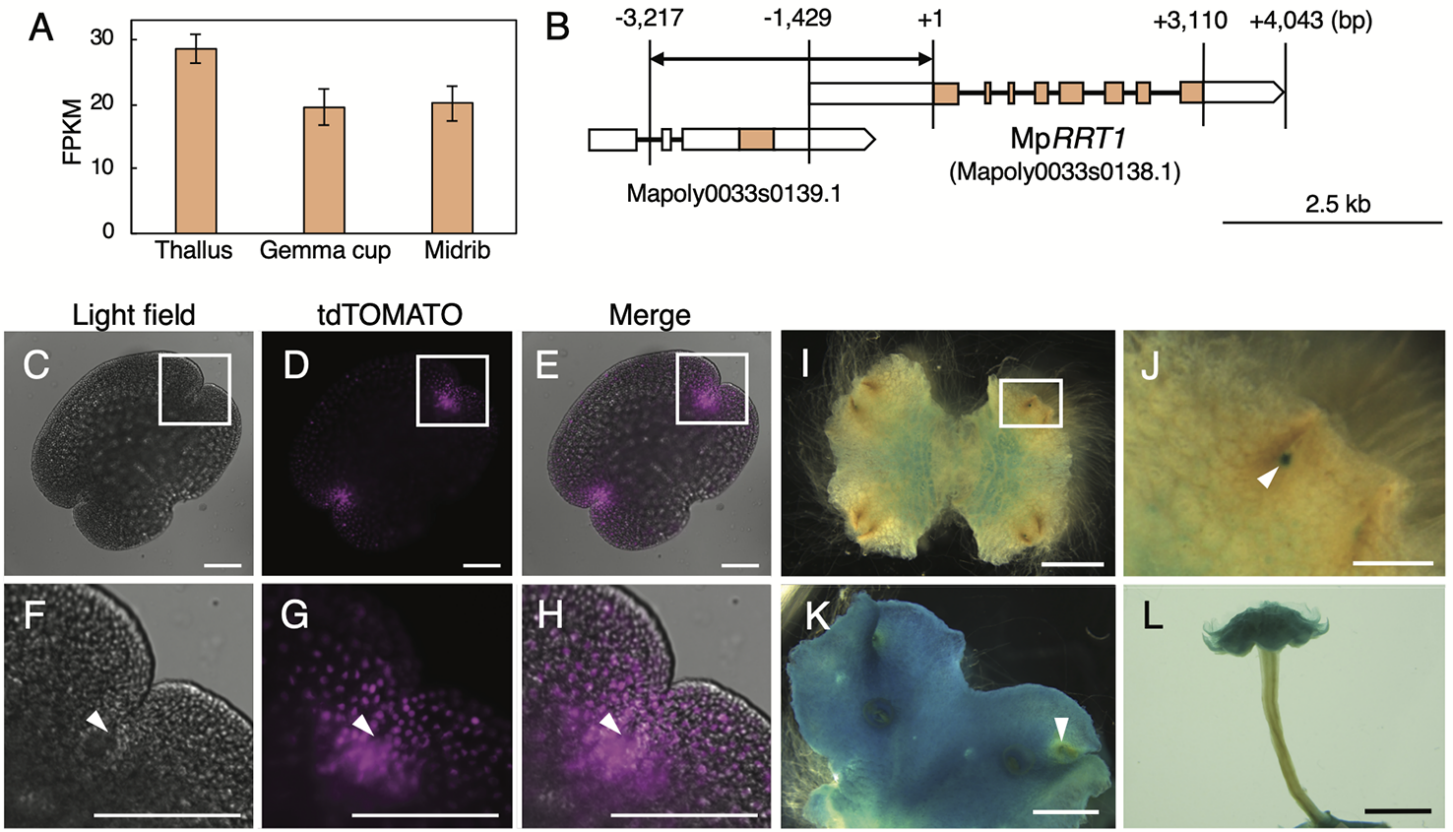
Expression profiles of Mp*RRT1* in liverwort. **(A)** Expression profile of Mp*RRT1* based on RNA-Seq data. Expression levels of Mp*RRT1* in the thallus, gemma cup, and midrib were estimated based on the relative abundances of transcripts with the unit fragments per kilobase million (FPKM). The values are presented as the mean of three biologically independent samples with SD. **(B)** Schematic representation of the structure of the Tak-1 Mp*RRT1* (Mapoly0033s0138.1) and the adjacent gene locus based on Marpolbase and sequencing results in this study. Orange and white boxes represent predicted exons and UTRs, respectively. Black lines represent introns. A bidirectional arrow indicates the promoter region used in reporter analyses. **(C–H)** Expression pattern of *pro*Mp*RRT1*:*tdTOMATO* in gemma. Images of light field **(C, F)**, fluorescence images of tdTOMATO **(D, G)**, and their merged images **(E, H)** are shown. Magnified images of the white squares in the upper panels are presented in the images in lower panels. White arrowheads indicate the apical notch. **(I–L)** GUS staining of *pro*Mp*RRT1*:*GUS* in 11-day-old thalli **(I**, **J),** 19-day-old thalli **(K)**, and antheridiophores **(L)**. **(J)** is a magnified image of the area of the white squares of **(I)**. White arrowhead indicates GUS staining at the apical notch. Scale bars, 100 μm **(C–H)**, 500 μm **(J)**, 2 mm **(I, K)**, and 5 mm **(L)**.

A previous RNA-seq analysis of *M. polymorpha* tissues (Bowman et al., 2017) showed that Mp*RRT1* was expressed in all tissues investigated (**Figure 4A**). To explore the expression pattern of Mp*RRT1* at a higher spatial resolution, we generated *promotor:tdTOMATO* and *promotor:GUS* lines for Mp*RRT1*. The predicted promotor region of Mp*RRT1* (*pro*Mp*RRT1*, 4,217 bp) was extracted from MarpolBase (http://marchantia.info). Although this region contained duplicated 1,000-bp nucleotide sequences (**Supplementary Figure S2**), we confirmed that this duplicated region was missing in the *M. polymorpha* wild-type genome by Sanger sequencing (**Supplementary Figure S3**). The structure of the Mp*RRT1* gene is shown in **Figure 4B**. The upstream promoter region (*pro*Mp*RRT*, 3,217 bp) was used for reporter analysis. According to MarpolBase, a predicted gene (Mapoly0033s0139.1) was located −0.7- to −3.9-kbp upstream of the Mp*RRT* gene (**Figure 4B**). We excluded the transcriptional start site of Mapoly0033s0139.1 (−3.9-kbp upstream) from the *pro*Mp*RRT1* region to avoid the unexpected transcription of this gene in transformants. Next, *pro*Mp*RRT1* was fused with the *tdTOMATO* gene (*pro*Mp*RRT1*:*tdTOMATO*) or *GUS* gene (*pro*Mp*RRT1*:*GUS*). Each resulting construct was then transformed into thalli of wild-type liverwort. The fluorescence of tdTOMATO was concentrated in the notch of the gemma (**Figures 4E, H**). Similarly, the strong signal at the apical notch was also observed by GUS-staining 11-day-old thalli in the *pro*Mp*RRT1*:*GUS* transformants (**Figures 4I, J**). In addition, GUS staining was observed in the region ~1 mm from the apical notch in 11-day-old thalli (**Figure 4I**). This region corresponds to the area in which thallus growth is decreased or ceases (Solly et al., 2017). The region of GUS staining was spread out in the 19-day-old thalli except for the region ~1 mm from the apical notch (**Figure 4K**). The region showing weak GUS staining was previously shown to correspond to the area exhibiting higher aerial growth rates than others (Solly et al., 2017). In the antheridiophore, strong GUS staining was observed in the lobed disc but not in the elongating stalk (**Figure 4L**). These expression patterns imply that Mp*RRT1* is predominantly expressed in the meristematic and maturation stage of development in liverwort tissues.

### Phenotype of the Mp*rrt1* Mutants

**Figure 5:**
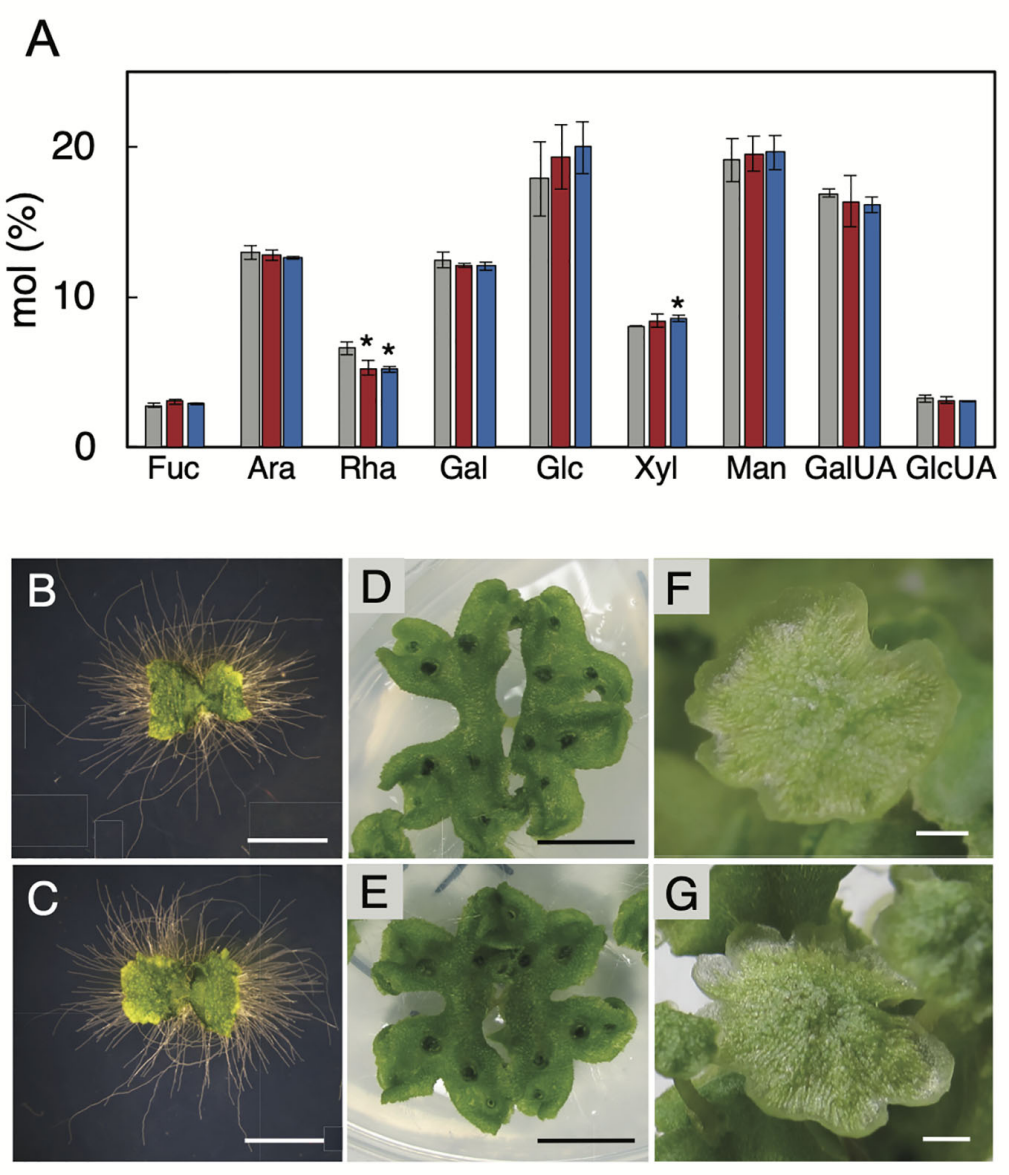
Chemotype and phenotype of the Mp*RRT1* genome-edited lines. **(A)** Monosaccharide compositions of thalli in the Mp*RRT1* genome-edited lines. Trifluoroacetic acid-hydrolysate non-cellulosic fractions from 21-day-old gemmalings of Tak-1 (gray), Mp*rrt1-1* (red), and Mp*rrt1-2* (blue) strains were subjected to ion-chromatography. The monosaccharide contents are shown as the percentages of the total amounts of monosaccharides. Values represent means of three biologically independent samples with SD. Asterisks indicate that the value was signiﬁcantly different from that of Tak-1 by Student’s t-test: *P < 0.05. Ara, arabinose; Rha, rhamnose; Fuc, fucose; Xyl, xylose; Man, mannose; Gal, galactose; Glc, glucose; GalUA, galacturonic acid; GlcUA, glucuronic acid. Nine-day-old gemmalings of Tak-1 **(B)** and the Mp*rrt1-1* mutant **(C)**. Gemmalings were grown on medium covered with cellophane to clearly observe rhizoids. Twenty-one-day-old gemmalings of Tak-1 **(D)** and the Mp*rrt1-1* mutant **(E)**. Antheridiophores of Tak-1 **(F)** and the Mp*rrt1-1* mutant **(G)**. Bars, 2 mm **(B, C, F, G)** and 1 cm **(D, E)**.

To investigate the significance of RG-I in *M. polymorpha* plants, we generated the Mp*RRT1*-deficient mutants by genome editing using the CRISPR/Cas9 system. These mutants were expected to be RG-I-deficient because Mp*RRT1* was considered the sole RG-I rhamnosyltransferase-encoding gene found in the *M. polymorpha* genome. We transformed a construct encoding a guide RNA that targeted the fourth exon of Mp*RRT1* (**Supplementary Figure S4A**) and detected >8 events of genome editing around the fourth exon of Mp*RRT1* in the genome at the T1 generation. Among them, we chose two mutants, Mp*rrt1-1* with a 155-bp deletion and a 16-bp insertion resulting in deletion of amino acids (ΔD158 to R188) (**Supplementary Figure S4B**) and Mp*rrt1-2* with a 5-bp deletion resulting in a frameshift that introduced a premature stop codon (**Supplementary Figure S4C**) for further analyses (**Supplementary Figure S5**). The percentages of rhamnose residues among cell wall monosaccharides of Mp*rrt1-1* and Mp*rrt1-2* were only 20% and 21% lower than wild-type levels, respectively (**Figure 5A**). There was also no significant difference in galacturonic acid contents between wild-type and the mutant strains (**Figure 5A**). We next investigated the morphological differences between the wild-type and the Mp*rrt1-1* mutant based on 9-day-old gemmalings, 21-day-old gemmalings, and antheridiophores. However, contrary to expectations, prominent morphological differences were not observed between wild-type and mutant strains (**Figures 5B–G**). These results suggested that Mp*RRT1* is not the sole *RRT* gene in *M. polymorpha* and that this species has other *RRT* genes in its genome.

### Gene Redundancy of *RRTs* in GT106

**Figure 6:**
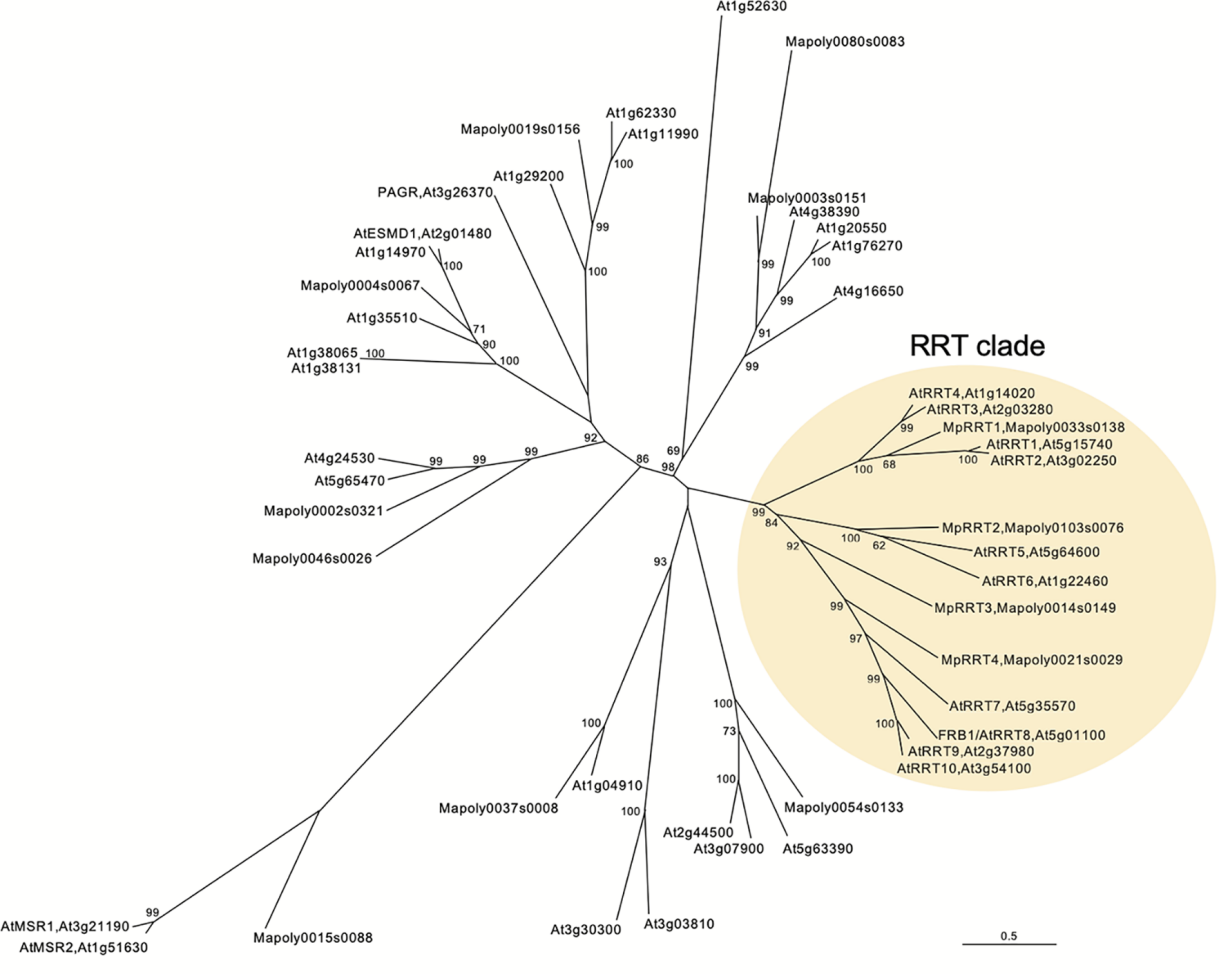
A neighbor joining phylogenetic tree of *Marchantia polymorpha* and *Arabidopsis thaliana* GT106 proteins. Amino acid sequences of 34 *Arabidopsis* and 13 *Marchantia* GT106 proteins were aligned using ClustalW, and bootstrap analyses were performed with 1,000 replicates. The RG-I rhamnosyltransferase (RRT) clade is highlighted in pale yellow. Scale bar indicates the number of amino acid substitutions per site.

**Figure 7:**
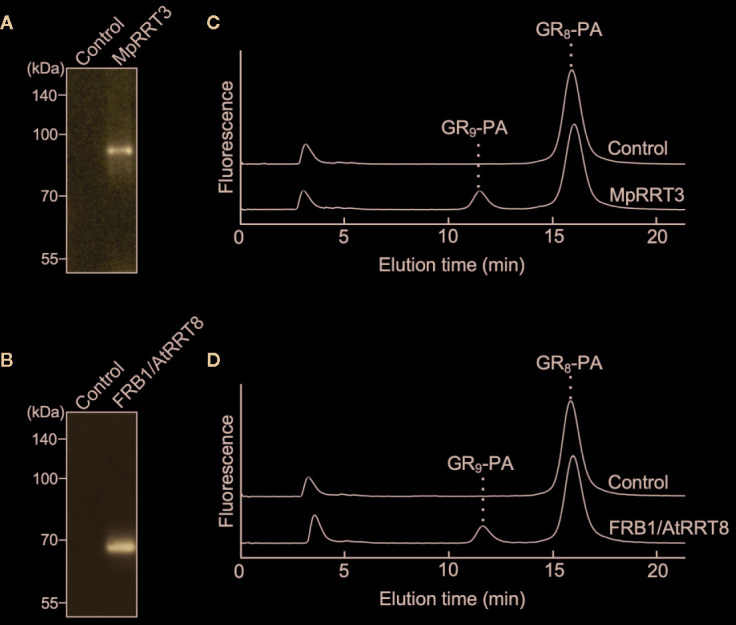
MpRRT3 and FRB1/AtRRT8 have RG-I rhamnosyltransferase activity. **(A, B)** SDS-PAGE of the recombinant **(A)** MpRRT3 and **(B)** FRB1/AtRRT8 proteins with a C-terminal FLAG tag expressed in tobacco leaves. The proteins were detected by immunoblotting using an anti-FLAG antibody. **(C, D)** Rhamnosyltransferase activity of **(C)** MpRRT3 and **(D)** FRB1/AtRRT8. The recombinant proteins were reacted with 50 µM GR_8_-PA and 0.5 mM UDP-Rha at 30°C for 1 h. The upper and lower chromatograms represent after enzyme reactions with the fraction from control proteins and the recombinant proteins.

Next, we examined the RG-I rhamnosyltransferase activity of GT106 proteins other than AtRRT1, AtRRT2, AtRRT3, AtRRT4 (Takenaka et al., 2018), and MpRRT1 (this study). First, we examined MpRRT3 (Mapoly0014s0149.1), which is most closely related to the clade containing the RRTs identified in the phylogenetic tree of GT106 (**Figure 6**). We expressed a recombinant MpRRT3 fused with a FLAG-tag as an 85-kDa protein, for which the molecular mass was calculated to be 81 kDa, in *N. benthamiana* leaves (**Figure 7A**). MpRRT3 exhibited the same RG-I rhamnosyltransferase activity as MpRRT1 (**Figure 7C**), as GR_8_-PA was used as an acceptor substrate.

The clade containing MpRRT3 in GT106 includes several *A. thaliana* putative glycosyltransferases (**Figure 6**). FRB1/AtRRT8, one *A. thaliana* putative enzyme, was shown to be involved in cell adhesion, but enzyme activity had not yet been characterized (Neumetzler et al., 2012). Accordingly, we produced a recombinant FRB1/AtRRT8 protein as a 68-kDa protein (calculated value, 72 kDa) in *N. benthamiana* leaves (**Figure 7B**) and detected its RG-I rhamnosyltransferase activity using GR_8_-PA as an acceptor substrate (**Figure 7D**).

These results extended the clade for pectin-synthetic RG-I rhamnosyltransferases in GT family 106. All proteins in the RRT clade defined as shown in **Figure 6** appear to have RG-I rhamnosyltransferase activity. Streptophyte plants have several RRT genes in their genomes (**Table 1**). *A. thaliana* (angiosperm, eudicot), *Oryza sativa* (angiosperm, monocot), *Picea abies* (gymnosperm), *Selaginella moellendorffii* (lycophyte), *M. polymorpha* (bryophyte), and *Klebsormidium flaccidum* (charophyte) were found to have 10, 11, 15, 7, 4, and 2 *RRT* genes in their genomes, respectively (**Table 1**).

**Table 1:** Gene numbers of GT106 and the RRT clade in plant genomes.

Taxonomic domains	Species	Gene number	
		GT106	RRT clade
Angiosperm (eudicot)	*Arabidopsis thaliana*	34	10
Angiosperm (monocot)	*Oryza sativa*	28	11
Gymnosperm	*Picea abies*	49	15
Lycophyte	*Selaginella moellendorffii*	19	7
Bryophyte	*Marchantia polymorpha*	13	4
Charophyte	*Klebsormidium flaccidum*	18	2

## Discussion

The first rhamnosyltransferase responsible for the biosynthesis of the RG-I backbone, named RRT, was identified in eudicot plants (Uehara et al., 2017; Takenaka et al., 2018). In this study, we demonstrated the existence of the same enzyme activity in the liverwort *M. polymorpha* using a recombinant MpRRT1 protein expressed in *N. benthamiana* (**Figure 1**). We also observed that the enzyme functions to produce RG-I *in vivo* (**Figure 2**). These results biochemically verified the evolutionary view that *RRT* exists in the genome of Streptophyta plants including the liverwort *M. polymorpha* (Takenaka et al., 2018).

Pectin biosynthesis occurs in the lumen of the Golgi apparatus (Driouich et al., 2012). Some RG-I biosynthetic glycosyltransferases have been shown to localize to the Golgi endomembrane system (Harholt et al., 2012; Liwanag et al., 2012; Takenaka et al., 2018). This study showed that MpRRT1 localizes to the *cis*-Golgi (**Figure 4**). The epitopes of LM5, which recognize the RG-I side chain galactan have been shown to be present mostly in the *trans*-Golgi and *trans*-Golgi network of flax root cells (Vicré et al., 1998). These results support the idea that the biosynthesis of the RG-I backbone and side chains occurs in early and late Golgi compartments, respectively.

The developmental (spatial and temporal) regulation of Mp*RRT1* expression was also observed (**Figures 3** and **4**). Reporter analysis suggested that Mp*RRT1* might function at the meristematic and maturation stage rather than the elongation phase in the development of thalli and antheridiophores (**Figure 4**). The Mp*rrt1*-deficient lines did not show prominent phenotypic changes (**Figure 5**), although this might require further detailed analysis. These results can be explained by the redundancy of *RRT* genes in the *M. polymorpha* genome (**Figure 6**). Each Mp*RRT* was expressed in all organs investigated (**Supplementary Figure S6**), showing that these glycosyltransferases cooperatively synthesize RG-I in each plant organ. This fits with the observation that the Mp*RRT1*-deficient mutants did not show complete elimination of RG-I in their cell walls. However, the biological significance of RG-I polysaccharides in *M. polymorpha* could not be solved in this study. This plant species still has a relative technological advantage over *A. thaliana* because of its lower redundancy with respect to RRTs (**Table 1**). The analysis of *M. polymorpha* mutants deficient in multiple *RRT* genes is the next subject for RG-I-related biology.

Streptophyte plants have several *RRT* genes in their genome (**Table 1**), meaning that approximately one-third of GT family106 exhibits RRT activity. The presence of a large number of *RRT* genes in the genomes of Streptophyta indicates that the evolution and diversification of RG-I biosynthesis was a critical event during the terrestrialization of Streptophyta.

The *A. thaliana* genome has 10 *RRT* genes in total (**Figure 6**). This number is comparable with that of GAUT (GT8), as 15 genes can be found in its genome (Atmodjo et al., 2013). GAUT is the enzyme responsible for biosynthesis of the HG backbone. This suggests that the biological significance of RG-I is similar to that of HG. The functional analysis of each GAUT has not been straightforward because of redundancy in this gene (Caffall et al., 2009). Therefore, it can be easily seen that the functional analysis of each *RRT* gene is difficult because all 10 RRT genes are expressed in all tissues and their expression levels and patterns are diverse based on the Arabidopsis eFP browser (Winter et al., 2007). However, *frb1*/At*rrt8* single-knockout mutations change the biochemical properties of the cell wall and middle lamella and affect cell-cell adhesion (Neumetzler et al., 2012). These pleiotropic effects of this mutation made it to be difficult to specify the glycosyltransferase activity of FRB1/AtRRT8. Taking the results of this study into account, RG-I synthesized by FRB1/AtRRT8 is involved in cell adhesion in the cell wall and/or middle lamella of *A. thaliana*. FRB1/AtRRT8 affects the contents of monosaccharide residues constituted of RG-I (Neumetzler et al., 2012). Further, it affects the abundance of arabinose and galactose residues rather than the reduction of rhamnose residues (Neumetzler et al., 2012). In this single *RRT*-deficient *A. thaliana*, the RG-I structure in the limited region associated with adhesion appears to be affected, and a reduction in rhamnose residues in the *frb1/*At*rrt8*-mutant could not be detected because other *RRTs* compensate for this to some extent. The changes in arabinose and galactose abundances in this mutant appear to be due to multifaceted effects caused by FRB1/AtRRT8 deficiency. Thus, pectin RG-I synthetic-RRTs are more redundant and diverse in GT106 (**Figure 6** and **Table 1**) than previously thought (Takenaka et al., 2018). A functional study of these RRTs will further contribute to the understanding of the biological roles of RG-I in land plants.

Approximately 30% of the enzymes in the GT106 family were found to be RRTs (**Figure 6** and **Table 1**). However, the biochemical characteristics of other enzymes belonging to the GT106 family appear to be quite different from those of RRTs. This is because previous mutant analyses of the genes belonging to GT106 have shown that they might be involved in the biosynthesis of other polysaccharides, including mannan (Stonebloom et al., 2016; Verger et al., 2016; Smith et al., 2018; Voiniciuc et al., 2019). These GT106 proteins would be undoubtedly glycosyltransferases, as their *in vivo* functions were lost due to substitutions of amino acid that appear to be catalytic residues (Smith et al., 2018; Voiniciuc et al., 2019). The next step for the study of GT106 is to detect glycosyltransferase activity and analyze biochemical characteristics for each enzyme.

In this study, we concluded that MpRRT1, MpRRT3, and FRB1/AtRRT8 have rhamnosyltransferase activity. This conclusion was highly probable because it was drawn from multiple lines of biochemical evidence (**Figures 1**, **2**, **5**, and **7**). However, we cannot completely exclude the possibility that the recombinant RRTs indirectly mediate rhamnosyltransferase activity because they were not completely purified from plant microsomal fractions. We did not show an image of the SDS-PAGE gel stained with Coomassie brilliant blue for the protein fractions used for glycosyltransferase assays but did show the western blotting pattern (**Figures 1** and **7**) because a protein band corresponding to the recombinant protein could not be detected by Coomassie brilliant blue staining. The question of whether RRT alone or a complex containing RRT has rhamnosyltransferase activity is an issue that needs to be clarified in the future.

We extended our knowledge of the RRTs involved in RG-I main chain biosynthesis. However, it has not been determined which galacturonosyltransferase is involved in biosynthesis of the RG-I main chain. Candidates for this include GATL5, GAUT11, and MUCI70, for which deletion mutants show severe phenotypes of reduced seed mucilage (Kong et al., 2013; Voiniciuc et al., 2018). Biochemical analysis of the enzymes encoded by these genes is eagerly awaited.

## Data Availability Statement

The raw data supporting the conclusions of this article will be made available by the authors, without undue reservation.

## Author Contributions

BW, TK, KN, and TI designed the research. BW, TK, YT, HK, SN, RY, and KI performed the research. BW, TK, YT, and KI analyzed the data. BW, TK, KN, and TI wrote the manuscript. All authors contributed to the article and approved the submitted version.

## Funding

This work was supported by a Grant-in-Aid for Scientific Research (No. 18H05495 and 19H03252 to TI, and 19K16173 to YT) from the Ministry of Education, Culture, Sports, Science, and Technology and a JICA Innovative Asia Scholarship (No. D1707016 to BW) from Japan International Cooperation Agency. It was also supported by the Mizutani Foundation for Glycoscience, the Novartis Foundation (Japan) for the Promotion of Science, and the Program for the Third-Phase R-GIRO Research from the Ritsumeikan Global Innovation Research Organization, Ritsumeikan University to TI.

## Conflict of Interest

The authors declare that the research was conducted in the absence of any commercial or financial relationships that could be construed as a potential conflict of interest.

## Abbreviations

Ara, arabinose; DP, degree of polymerization; FPKM, fragments per kilobase million; Fuc, fucose; Gal, galactose; GalUA, galacturonic acid; Glc, glucose; GlcUA, glucuronic acid; HG, homogalacturonan; Man, mannose; Rha, rhamnose; RG-I, rhamnogalacturonan I; RRT, RG-I rhamnosyltransferase; Xyl, xylose.
