# Supplementary material for: Diversity of Pectin Rhamnogalacturonan I Rhamnosyltransferases in Glycosyltransferase Family 106
Source: Front Plant Sci. 2020 Jul 2;11:997. doi: 10.3389/fpls.2020.00997 (PMC7343896; doi:10.3389/fpls.2020.00997)
Supplement: Supplementary file 1 [file DataSheet_1.pdf]

Supplementary Table 1. Nucleotide sequences of oligonucleotides used in this study.

Primer name	Sequence
MpRRT1-FLAG_F	ACGGGGGACTCTAGAATGCTCAAGGGGGGAATAC
MpRRT1-FLAG_R	GGGAAATTCGAGCTCTTACTTGTCGTCATCGTCCTTGTAGTCCTTGAAGGAAAAA TTCCG
MpRRT3-FLAG_F	ACACGGGGGACTCTAGAATGGACAGGGGCGAC
MpRRT3-FLAG_R	ATCGGGGAAATTCGAGCTCTTACTTGTCGTCATCGTCCTTGTAGTCTTGCGTGGTG GTGC
MpRRT1_F	GCTAAGACCCTCGAGATGCTCAAGGGGGGAATAC
MpRRT1_R	AACTCTAGAACTAGTTCACTTGAAGGAAAAATTCCGCGAG
MpRRT1_CR1_F	CTCGATGCCAGGATGGTAGCCGAG
MpRRT1_CR1_R	AAACCTCGGCTACCATCCTGGCAT
MpRRT1_CR_seqF1	TCACCTTACTCCCCTTCCAGTA
MpRRT1_CR_seqF2	GTGCGTGAGAAGCAGTAATGTC
MpRRT1_CR_seqR	GCACCAATCTTCGACCTAATTC
MpRRT1-4217_F	CACCCAATTGGGCCTTGAGGTGTAAGCGC
MpRRT1_0_R	GGCTCCTGTCTTCCGAGTTCGGGTA
MpRRT1_1_F	CACCATGCTCAAGGGGGGAATACCGAGTC
MpRRT1+3107_nosto p_R	CTTGAAGGAAAAATTCCGCGAGTAG
AtRRT8-FLAG _F	ACGGGGGACTCTAGAATGTCAGTCGGCGTTCCAG
AtRRT8-FLAG _R	GGGAAATTCGAGCTCTTACTTGTCGTCATCGTCCTTGTAGTCTCTCAGAGATTGTG CTCGTAG

Supplementary Table 2. Abbreviations and structures of RG-I oligosaccharides used in this study.

Abbreviation	Structure
RG_3-PA	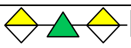 PA
GR_4-PA	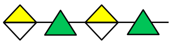 PA
RG_5-PA	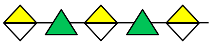 PA
GR_6-PA	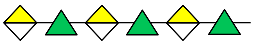 PA
GR_8-PA	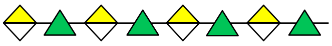 PA
GR_{10}-PA	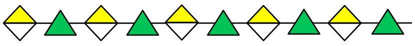 PA
GR_{12}-PA	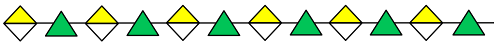 PA
GR_{14}-PA	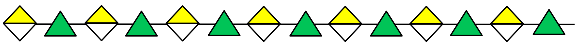 PA

Green triangles, yellow divided diamonds, and PA represent rhamnose residue, galacturonic acid residue, and fluorescent pyridylamino group, respectively.

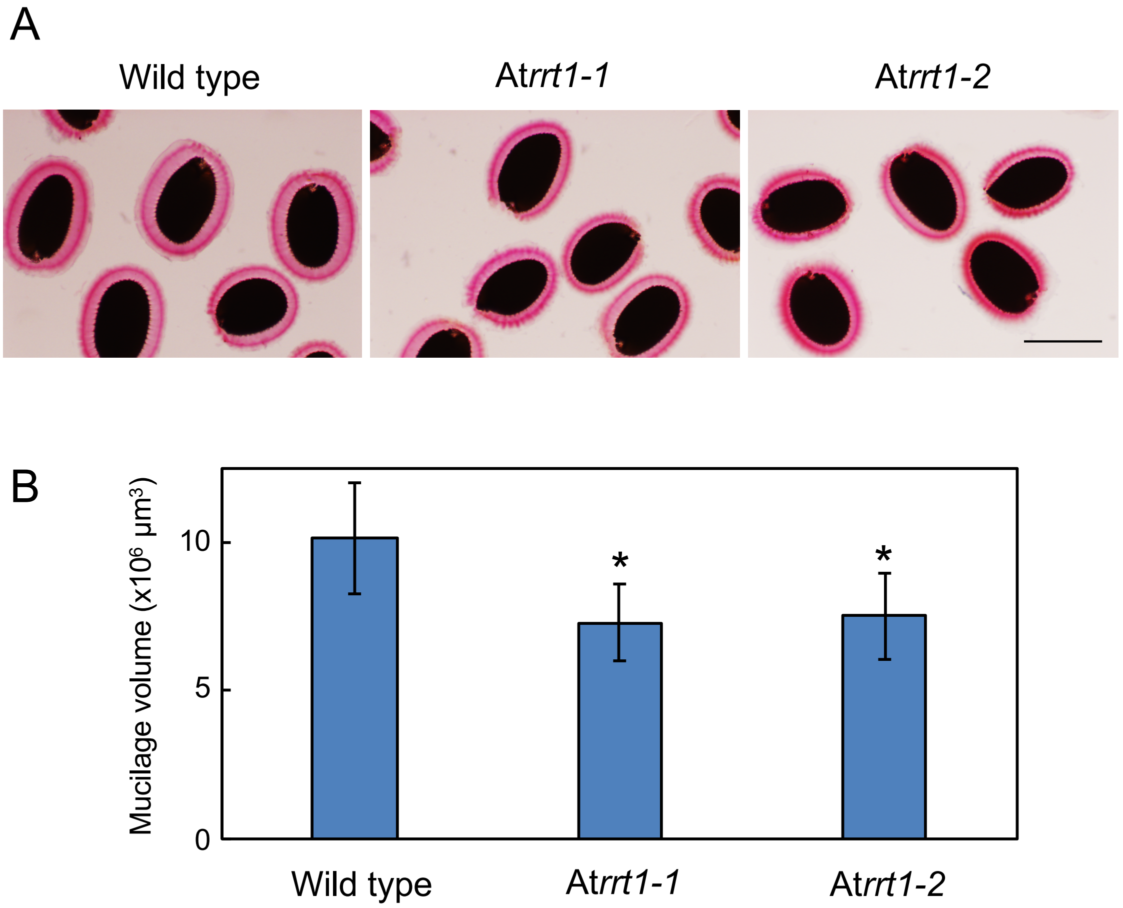

Supplementary Figure 1. Seed coat mucilage phenotype of At*rrt1*. (A) *Arabidopsis* seed coat mucilage of the wild-type, At*rrt1-1* (SALK_022924), and At*rrt1-2* (SALK_042968) strains, stained with ruthenium red. Scale bar, 250 µm. **(B)** Mucilage volumes of seed coats in the wild-type, At*rrt1-1*, and At*rrt1-2* strains. The mucilage volumes are presented as the mean values of 20 seeds with SD. Asterisk indicates that the value was significantly different from those of the wild type and two At*rrt1* mutant strains by Student's t-test: $*P < 0.05$.

>pMpRRT_Malpolbase (4,217 bp)
CAATTGGGCCTTGAGGTGTAAGCGCTCTCGATCTTCGCCTGACTTTGGGTGTGGCCGAGGTAGCTCCACATTTCTGCGCGATCAGCTCGGCCTCTGGACCGGGAATCATATTTGTAAGAGA
ATTCCTTTTCCTGCTTGATCTGACTTGTCTGTGAGATGGCTTTTTCAGAGTGAAGTTCCGCTCCGAGCTCGTCGAAAGCAGATTTTGAGGAGTTTTGTTTCGCGCTGCAGCTGTCAATTGG
TTCCCTCGTGCTCTGTATTTGGAATTTCGTCTCATCCTGCTTGGCCAGGTACGGTACATGTTTTGCTTCCCAAACTCCTTGATGTATTTGGCCATTGTCTCATCGGCTGGTCAGAATCTTC
TCAACAGATGCTTTCTTCAGGCCAGGCTGAAGAGTCTCCGCAACATCTCCAGAAGTCTAGAGGTCGACATCTGTCATAGATGAGCTTCAATACAAAACTTCCTACAGTTTCTACTTTGGGA
GACAAAGCTTCTCCTCGTCGATGCTTCACACTCGCATCTTTGATGTAGTCGAGGTACCTAGGCTTATTGCCCGGAGAAAGGAAGGCCCAAGGTCTAACCTCACATCCGATACTTGTTCGAC
AGCTGCTCCGGTATCTTCCGAGAAACTTGAGAAAACTCACTGGAATCCATGGTTGCATCGTAGGCAGTACCTTTCTCGACGAGTTAAGTGATGTCACCAGGGAGAACTCTTTCCGTTGAAT
CATTGCAACTGTTGTGTACATGACGATTGAAGCATAAGACTGCTGCATGATGAGACGAGATTCTCCTCTATTTTGTATGTTGCAGCATCCGATCATTCCAGTCCAGCTCAGCCCGTTGACA
CTTCTTCAAATTTGCAAGCAAACTCTTCATGTGCAAGCAACACCCTCTCGCTCTCTCTCTCTCTTACTCGAGTGCATGAGCGGCGGTGACTGTACCGGATGGCATTTCGTTAGAAGAGG
GTTCCGTGACCTCGGGCACGCAGCGAGGGCAGGCGGAGTCGAGTCCAGTCACCAGTGGCTGGGCTCTTCGGGGCCGTAGGATGCTCCCATCGTATACGCAAAAGGACCATACAGTACAGTA
TGTCGCATCGTATGATGTAGCAGGCAGCCAGCGAGCCACTTGCGGAGGAGGAAGTGCGTCACTTCCGTCGCCAAGCCCCAACGAGCCCTGCGCTCTGGGCCTATCCGCTCCTCCATATAAG
GAGAAGCTCTGTTCCCTCGGCGTGTCGTTTGGCTCCAGCTTTGGACATCCACAAATAGAGCCTCGCTGTCGGGACTGCTCGCTGGCTCCGACGCCAGCTTGGCGTCGCGCTATTTTACCCG
CGACGGCGTGGGTAGGTGCTTGGTCACACGCGGGCCCTCTCTCGTCAGCAGAGCACGCAGCCGGTGGCTTGCATTACGAAGTATACCATTGGCCCTGGGGCTCAAGGTCCCGCCGATGGCA
GTCTTCGTCGCGCTAGTCGATCCGTGGCCTCGAACCATCTCGTCGCATGATTGCGACGTCGGTGGAGAGAGGAGGCTCTGACAGCGCGACGGCGCCTACTGGCACGAGGGAGACGAGACGA
GCATTACCGTTCCGGCATGTCTGCCTGCGAGCGAGAGCCGGGCAAAGGTCGAGGGCGAGAGTGGCCGAGGGGAAAGGACGGCGGGCAAATTCGGGGGCGAGATGAGGATGCATGTCGTGTG
TCAGAGGAGAGGACCGACTCCCTCAGAAAGATGTAGGCGCATGAGAAGAATCCGATAAGAATGTCGACCGTCAGATGCGCACATCGTCGGCCGCTTTGAGCGGAGTCTGCTTGCGTGCGCA
GCCATCGTCGGATGCGCGAATTGACAGTGAAGATGGAGTTGCCAGATTCTTTCTATCTGAACAGAGGATTGTGAACAGCGCAGGGCAGTGCAGTGCAGACGACGACGACGACGACGACGAC
TGGCGCAGGGCGACCAAGGAGTGAGCGGAAATTGCCGTTAACTTAGCCCGGCAGAATTGGAGTGGTCGGTCTTTCATGTCGAGACATCGAAACCAGCGGAGCTGCAGGAGGACCCAGAACA
AGTGGGTGTGGGGTTCCTGACTGTGGTCGAGCATCAGTAGTGCCACTGTGCGGATTGTACCATCAGCACAAGTAGAGGCGGAGGAGGAGGAGGAGGATGCGGAGGTAGTAGTTCTAGTCGT
AGAAGAGAAGAGAGGAGTAGAAGTAGCAGTAGCTGTAGTGATGATGAATGAGAGGAGTGCAGCTGGGCCGACGACGATGGTCCTGTGAAGACTCTTCAGAACAGCGAGAAGCAGAGAGAGA
GAGAGAGAGAGAGAGAGAGCAAGAGAGGTAAACCGAGGCAGGCAGGCAGTGAACGGATTGTCGCATGTTTTGACGACCATGGCAAGAGAGACACGGACGGAGCGCATGTGAGGGAGGGAGG
GACGAAGGGAGGAGCAGGAGGCAGGGAGAGAGGTGTGCACTGCATTGTTCCTCTGCCGCTGCTGCTGTAGAGTAGAGTAGAGAAGCGACGAGAAGACGAAAGGGGCAACACAGGGGCCAGC
GAGTCTAGTGTGCTTGTTGTTGTCAGTGGCAGCCTTCATTTCGGACAATAGCGAGGAGAAGGGGAGAGAGAGAGAGAGAGAGAGAGAGAGAGAGAGAGAGAGAGAGAGAGAGAGAGAGA
GAGAGAGAGAGAGAGAGAGAGAGAGAGGTGTGTCAGAGGAGAGGACCGACTCCCTCAGAAAGATGTAGGCGCATGAGAAGAATCCGATAAGAATGTCGACCGTCAGATGCGCACATCGTCG
GCCGCTTTGAGCGGAGTCTGCTTGCGTGCGCAGCCATCGTCGGATGCGCGAATTGACAGTGAAGATGGAGTTGCCAGATTCTTTCTATCTGAACAGAGGATTGTGAACAGCGCAGGGCAGT
GCAGTGCAGACGACGACGACGACGACGACGACTGGCGCAGGGCGACCAAGGAGTGAGCGGAAATTGCCGTTAACTTAGCCCGGCAGAATTGGAGTGGTCGGTCTTTCATGTCGAGACATCG
AAACCAGCGGAGCTGCAGGAGGACCCAGAACAAGTGGGTGTGGGGTTCCTGACTGTGGTCGAGCATCAGTAGTGCCACTGTGCGGATTGTACCATCAGCACAAGTAGAGGCGGAGGAGGAG
GAGGAGGATGCGGAGGTAGTAGTTCTAGTCGTAGAAGAGAAGAGAGGAGTAGAAGTAGCAGTAGCTGTAGTGATGATGAATGAGAGGAGTGCAGCTGGGCCGACGACGATGGTCCTGTGAA
GACTCTTCAGAACAGCGAGAAGCAGAGAGAGAGAGAGAGAGAGAGAGAGAGAGCAAGAGAGGTAAACCGAGGCAGGCAGGCAGTGAACGGATTGTCGCATGTTTTGACGACCATGGCAAGAGA
GACACGGACGGAGCGCATGTGAGGGAGGGAGGGACGAAGGGAGGAGCAGGAGGCAGGGAGAGAGGTGTGCACTGCATTGTTCCTCTGCCGCTGCTGCTGTAGAGTAGAGTAGAGAAGCGAC
GAGAAGACGAAAGGGGCAACACAGGGGCCAGCGAGTCTAGTGTGCTTGTTGTTGTCAGTGGCAGCCTTCATTTCGGACAATAGCGAGGAGAAGGGGAGAGAGAGAGAGAGAGAGAGAGAGA
GAGAGAGAGAGAGAGAGAGAGAGAGAGAGAGAGAGAGAGAGAGAGAGAGAGAGAGAGAGAGAGAGAGGATAATGGTGATGGATTGCAGGGTGCGGCCGTGGGGGATTGGGCTCGGCAGACACAATACAG
TGTGCGTTTCTTGTGGATGAGAGCAGATATGTGGTGGCAGCAGGCAGGCGGAGGCGATAACGTATAATGCAGCTGAAAGCATAAGCCAGGATCAGATTAATTGGTACACAGAGCCCGTGCG
GCGACTGTGGACGATCGCCCTCTGTGTAGTGGTCGAGGTAGTTGCCTGGACTAGATCCTCACCAATTGATGCTCGGAGGAATTAGTCCTCGGACGCGAGCAGGAACAGGTTCTGTCCAGCA
TCCCTGGACCTTGATCCGCGACACGGGCTTGGCCCCACACATTGCCTCGGATTGCCGACACTCGGTTTTGTAAAGATTTTGGAATCTCCCCGCTCGTACATCCGCTGCGACTTCTCGATGA
CCCGACGCGTGCTTCGACGCTTTATGGTGGATGCCTAATTTGAAGATTTTCATTGGAGGGGGGGTATGCAGCGGTTATTACCCGAACTCGGAAGACAGGAGCC

>pMpRRT (3,217 bp)
CAATTGGGCCTTGAGGTGTAAGCGCTCTCGATCTTCGCCTGACTTTGGGTGTGGCCGAGGTAGCTCCACATTTCTGCGCGATCAGCTCGGCCTCTGGACCGGGAATCATATTTGTAAGAGA
ATTCCTTTTCCTGCTTGATCTGACTTGTCTGTGAGATGGCTTTTTCAGAGTGAAGTTCCGCTCCGAGCTCGTCGAAAGCAGATTTTGAGGAGTTTTGTTTCGCGCTGCAGCTGTCAATTGG
TTCCCTCGTGCTCTGTATTTGGAATTTCGTCTCATCCTGCTTGGCCAGGTACGGTACATGTTTTGCTTCCCAAACTCCTTGATGTATTTGGCCATTGTCTCATCGGCTGGTCAGAATCTTC
TCAACAGATGCTTTCTTCAGGCCAGGCTGAAGAGTCTCCGCAACATCTCCAGAAGTCTAGAGGTCGACATCTGTCATAGATGAGCTTCAATACAAAACTTCCTACAGTTTCTACTTTGGGA
GACAAAGCTTCTCCTCGTCGATGCTTCACACTCGCATCTTTGATGTAGTCGAGGTACCTAGGCTTATTGCCCGGAGAAAGGAAGGCCCAAGGTCTAACCTCACATCCGATACTTGTTCGAC
AGCTGCTCCGGTATCTTCCGAGAAACTTGAGAAAACTCACTGGAATCCATGGTTGCATCGTAGGCAGTACCTTTCTCGACGAGTTAAGTGATGTCACCAGGGAGAACTCTTTCCGTTGAAT
CATTGCAACTGTTGTGTACATGACGATTGAAGCATAAGACTGCTGCATGATGAGACGAGATTCTCCTCTATTTTGTATGTTGCAGCATCCGATCATTCCAGTCCAGCTCAGCCCGTTGACA
CTTCTTCAAATTTGCAAGCAAACTCTTCATGTGCAAGCAACACCCTCTCGCTCTCTCTCTCTCTTACTCGAGTGCATGAGCGGCGGTGACTGTACCGGATGGCATTTCGTTAGAAGAGG
GTTCCGTGACCTCGGGCACGCAGCGAGGGCAGGCGGAGTCGAGTCCAGTCACCAGTGGCTGGGCTCTTCGGGGCCGTAGGATGCTCCCATCGTATACGCAAAAGGACCATACAGTACAGTA
TGTCGCATCGTATGATGTAGCAGGCAGCCAGCGAGCCACTTGCGGAGGAGGAAGTGCGTCACTTCCGTCGCCAAGCCCCAACGAGCCCTGCGCTCTGGGCCTATCCGCTCCTCCATATAAG
GAGAAGCTCTGTTCCCTCGGCGTGTCGTTTGGCTCCAGCTTTGGACATCCACAAATAGAGCCTCGCTGTCGGGACTGCTCGCTGGCTCCGACGCCAGCTTGGCGTCGCGCTATTTTACCCG
CGACGGCGTGGGTAGGTGCTTGGTCACACGCGGGCCCTCTCTCGTCAGCAGAGCACGCAGCCGGTGGCTTGCATTACGAAGTATACCATTGGCCCTGGGGCTCAAGGTCCCGCCGATGGCA
GTCTTCGTCGCGCTAGTCGATCCGTGGCCTCGAACCATCTCGTCGCATGATTGCGACGTCGGTGGAGAGAGGAGGCTCTGACAGCGCGACGGCGCCTACTGGCACGAGGGAGACGAGACGA
GCATTACCGTTCCGGCATGTCTGCCTGCGAGCGAGAGCCGGGCAAAGGTCGAGGGCGAGAGTGGCCGAGGGGAAAGGACGGCGGGCAAATTCGGGGGCGAGATGAGGATGCATGTCGTGTG
TCAGAGGAGAGGACCGACTCCCTCAGAAAGATGTAGGCGCATGAGAAGAATCCGATAAGAATGTCGACCGTCAGATGCGCACATCGTCGGCCGCTTTGAGCGGAGTCTGCTTGCGTGCGCA
GCCATCGTCGGATGCGCGAATTGACAGTGAAGATGGAGTTGCCAGATTCTTTCTATCTGAACAGAGGATTGTGAACAGCGCAGGGCAGTGCAGTGCAGACGACGACGACGACGACGACGAC
TGGCGCAGGGCGACCAAGGAGTGAGCGGAAATTGCCGTTAACTTAGCCCGGCAGAATTGGAGTGGTCGGTCTTTCATGTCGAGACATCGAAACCAGCGGAGCTGCAGGAGGACCCAGAACA
AGTGGGTGTGGGGTTCCTGACTGTGGTCGAGCATCAGTAGTGCCACTGTGCGGATTGTACCATCAGCACAAGTAGAGGCGGAGGAGGAGGAGGAGGATGCGGAGGTAGTAGTTCTAGTCGT
AGAAGAGAAGAGAGGAGTAGAAGTAGCAGTAGCTGTAGTGATGATGAATGAGAGGAGTGCAGCTGGGCCGACGACGATGGTCCTGTGAAGACTCTTCAGAACAGCGAGAAGCAGAGAGAGA
GAGAGAGAGAGAGAGAGAGCAAGAGAGGTAAACCGAGGCAGGCAGGCAGTGAACGGATTGTCGCATGTTTTGACGACCATGGCAAGAGAGACACGGACGGAGCGCATGTGAGGGAGGGAGG
GACGAAGGGAGGAGCAGGAGGCAGGGAGAGAGGTGTGCACTGCATTGTTCCTCTGCCGCTGCTGCTGTAGAGTAGAGTAGAGAAGCGACGAGAAGACGAAAGGGGCAACACAGGGGCCAGC
GAGTCTAGTGTGCTTGTTGTTGTCAGTGGCAGCCTTCATTTCGGACAATAGCGAGGAGAAGGGGAGAGAGAGAGAGAGAGAGAGAGAGAGAGAGAGAGAGAGAGAGAGAGAGAGAGAGA
GAGAGAGAGAGAGAGAGAGAGAGAGAGGATAATGGTGATGGATTGCAGGGTGCGGCCGTGGGGGATTGGGCTCGGCAGACACAATACAGTGTGCGTTTCTTGTGGATGAGAGCAGATATGT
GGTGGCAGCAGGCAGGCGGAGGCGATAACGTATAATGCAGCTGAAAGCATAAGCCAGGATCAGATTAATTGGTACACAGAGCCCGTGCGGCGACTGTGGACGATCGCCCTCTGTGTAGTGG
TCGAGGTAGTTGCCTGGACTAGATCCTCACCAATTGATGCTCGGAGGAATTAGTCCTCGGACGCGAGCAGGAACAGGTTCTGTCCAGCATCCCTGGACCTTGATCCGCGACACGGGCTTGG
CCCCACACATTGCCTCGGATTGCCGACACTCGGTTTTGTAAAGATTTTGGAATCTCCCCGCTCGTACATCCGCTGCGACTTCTCGATGACCCGACGCGTGCTTCGACGCTTTATGGTGGAT
GCCTAATTTGAAGATTTTCATTGGAGGGGGGGTATGCAGCGGTTATTACCCGAACTCGGAAGACAGGAGCC

Supplementary Figure 2. Mp*RRT1* promoter sequence in the Tak-1 genome. *pro*Mp*RRT1*_MarpolBase (4,217 bp) is the sequence extracted from the MarpolBase {scaffold_33: 1,349,919.. 1,354,136 (- strand)}. *pro*Mp*RRT1* (3,217 bp) is the sequence confirmed by Sanger sequencing and used in reporter analyses in this study (−3,217 to −1). Duplicated 1,000-bp sequences are highlighted in yellow and sky blue. Red sequences indicate the 5′UTR region based on the MarpolBase. The *Nco*I sites used in Supplementary Figure 3 are indicated with rectangles.

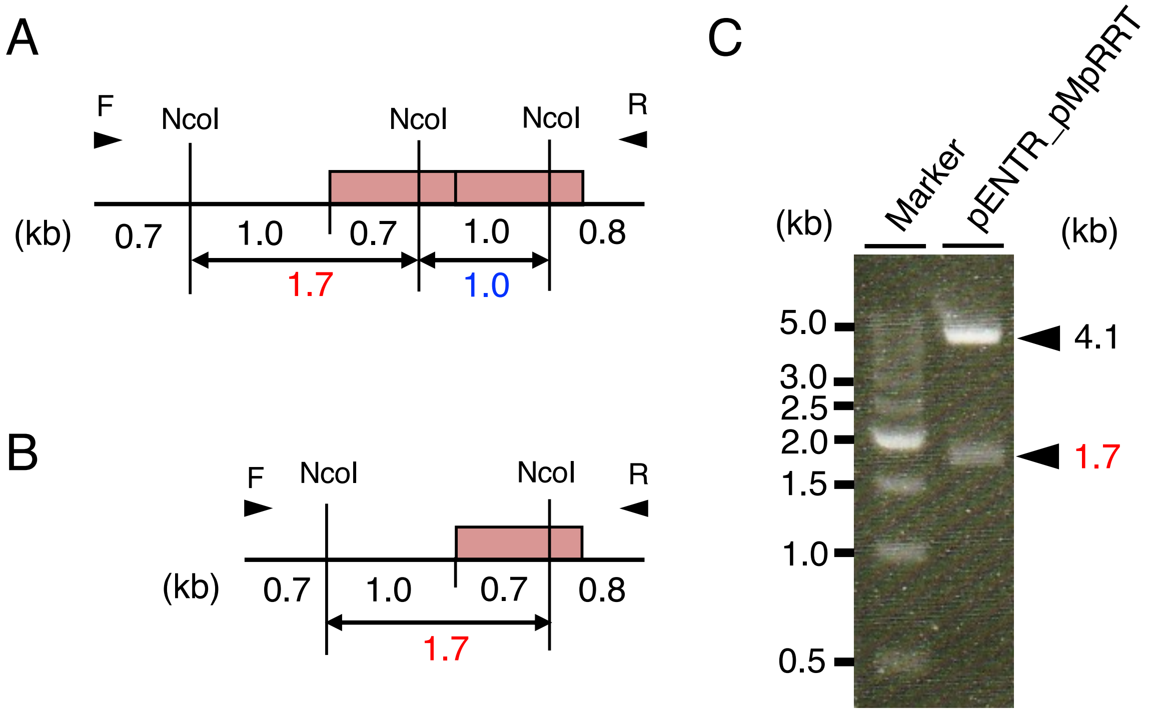

Supplementary Figure 3. The 1.0-kbp sequence of the Mp*RRT1* promoter is not tandemly duplicated in Tak-1. (A, B) Schematic representation of the Mp*RRT1* promoter based on MarpolBase **(A)** and this study **(B)**. The expected fragment sequences amplified with primers F (MpRRT1-4217_F) and R (MpRRT1_0_R) (Supplementary Table 1) are shown as arrowheads. Mp*RRT1* promoter sequence of MarpolBase is derived from scaffold_33: 1,349,919..1,354,136 (- strand). Orange rectangles indicate the 1.0-kbp sequence tandemly duplicated in MarpolBase. The positions of recognition sites for *Nco*I restriction enzyme are shown. Expected fragment sizes after digestion with *Nco*I are shown by bidirectional arrows. **(C)** DNA fragments of pENTR_pMpRRT1 digested with restriction enzyme *Nco*I. Based on the length of the pENTR/D_TOPO plasmid (2.6 kb), expected fragments for **(A)** and **(B)** are 4.1/1.7/1.0 kb and 4.1/1.7 kb, respectively. No 1.0-kb fragment indicates the absence of the 1.0-kbp duplication.

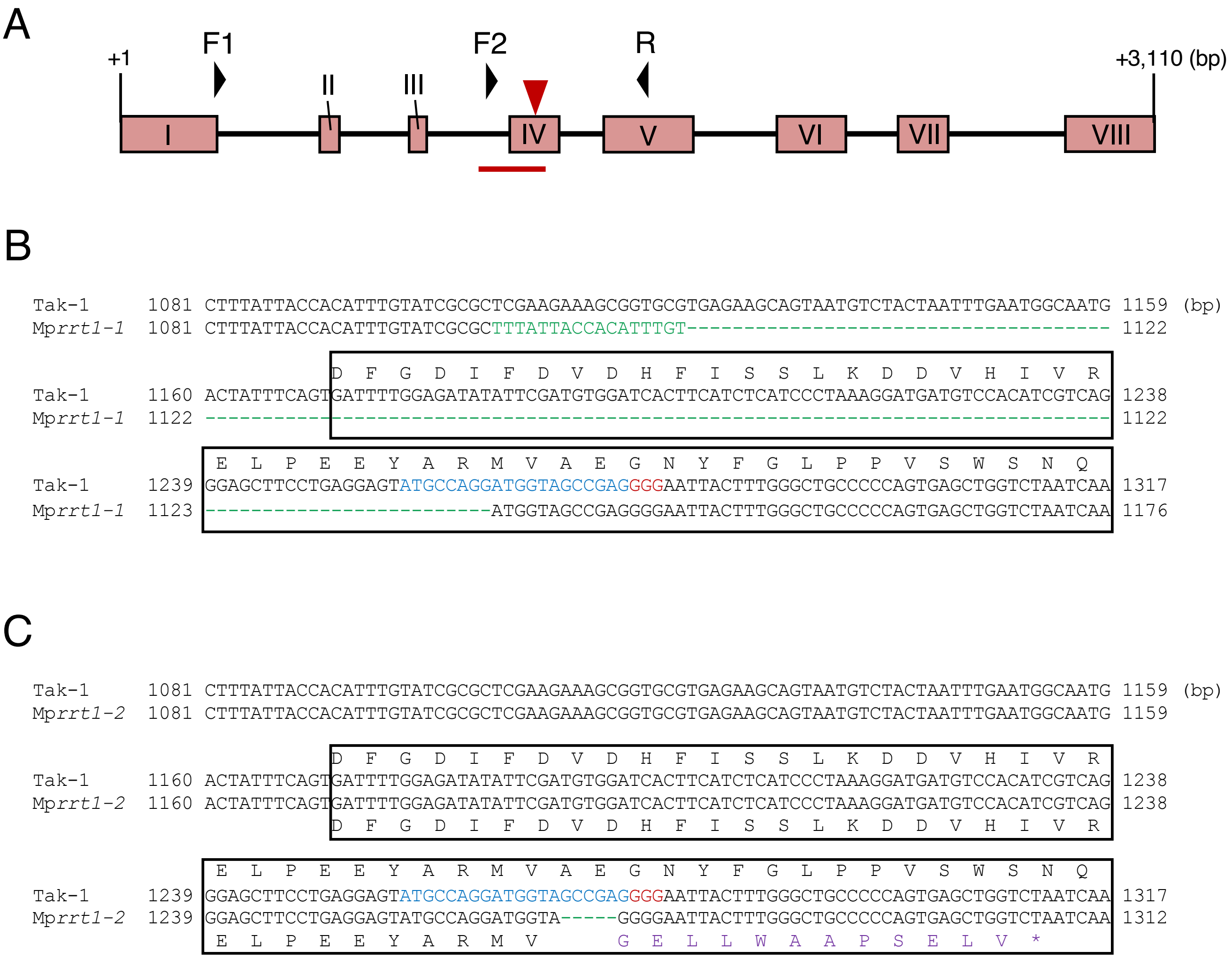

Supplementary Figure 4. Construction of the Mp*RRT1* genome-edited lines. (A) Schematic representation indicating positions of guide RNAs for the genome editing of Mp*RRT1*. Orange boxes and black lines represent predicted exons and introns, respectively. Roman numerals represent exon numbers. A red arrowhead indicates the position of the guide RNA for the genome editing of Mp*RRT1*. F1, F2, and R represent primers used to confirm genome editing (Supplementary Figure 5). **(B, C)** Comparison of Mp*RRT1* genomic sequences in the Tak-1 and Mp*RRT1* genome-edited lines, Mp*rrt1-1* **(B)** and Mp*rrt1-2* **(C)**. Nucleotide sequences of the red line in A are shown. The squares indicate the sequences of the 4th exon. The Tak-1 sequence is shown together with the PAM sequence (red) and the guide RNA (blue). The amino acid sequences encoded by Tak-1 and Mp*RRT1* genome-edited lines are shown at the top and bottom of their nucleotide sequences, respectively. Mp*rrt1-1* had a 155-bp deletion and a 16-bp insertion at the 3rd intron and 4th exon, respectively (B; green) resulting in deletion of amino acids (ΔD158 to R188). Mp*rrt1-2* had a 5-bp deletion (C; green) resulting in a frameshift that introduced a premature stop codon (purple).

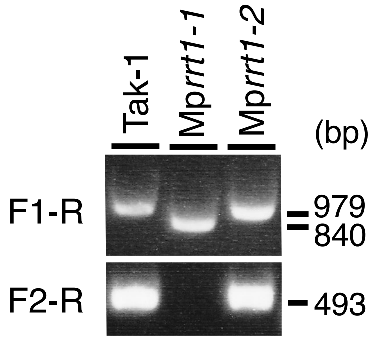

Supplementary Figure 5. Genotype of the Tak-1 and Mp*RRT1* genome-edited lines. Primer combinations are shown on the left. F1, F2, and R represent primers MpRRT1_CR_seqF1, MpRRT1_CR_seqF2, and MpRRT1_CR_seqR (Supplementary Table 1), respectively. The positions of each primer in Mp*RRT1* are shown in Supplementary Figure 4A. The sizes of the PCR products from Tak-1 (979 bp) and Mp*rrt1-1* (840 bp) are indicated on the right.

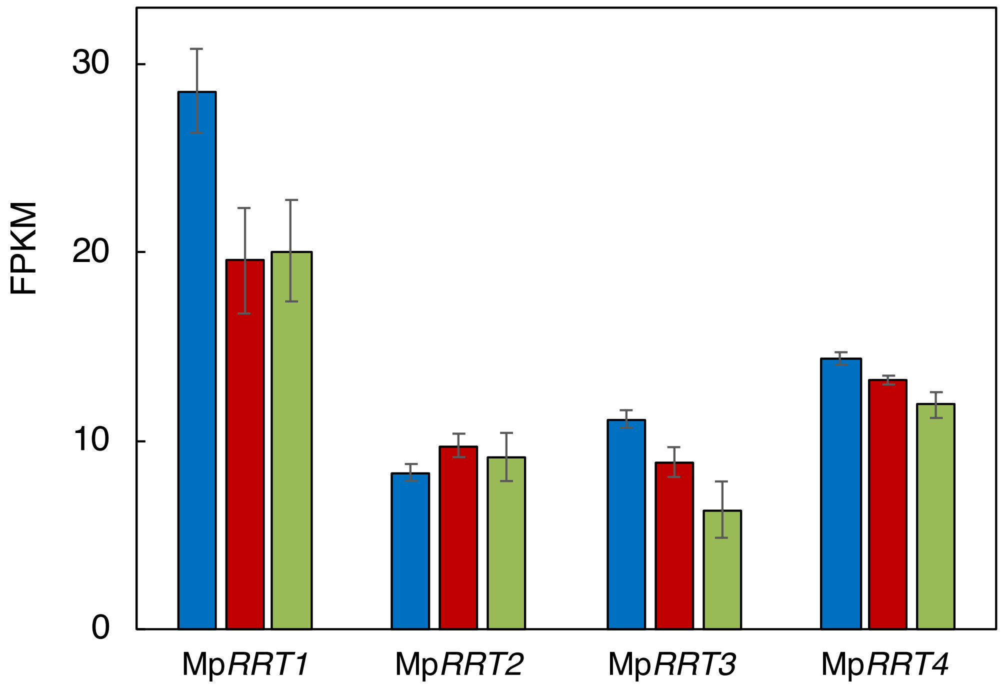

Supplementary Figure 6. Expression profiles of Mp*RRTs* from composite RNA-Seq data. The expression levels of four Mp*RRTs* in thallus (blue), gemma cup (red), and midrib (green) were estimated based on the relative abundances of transcripts with the unit fragments per kilobase million (FPKM). The values are presented as the mean values of three biological replicates with SD.
